# Expected and verified benefits from old and new corticosteroid treatments in IgA nephropathy: from trials in adults to new IPNA-KDIGO guidelines

**DOI:** 10.1007/s00467-025-06725-1

**Published:** 2025-03-05

**Authors:** Licia Peruzzi, Rosanna Coppo

**Affiliations:** 1 Pediatric Nephrology Unit, Regina Margherita Children’s Hospital, University of Turin, AOU Città della Salute e della Scienza di Torino, Piazza Polonia 94, 10126 Turin, Italy; 2 Fondazione Ricerca Molinette, Turin, Italy

**Keywords:** IgA nephropathy, Children, Treatment, Glucocorticosteroids, Budesonide intestinal release formulation

## Abstract

IgA nephropathy in children has a potential progression risk over decades of adult life. For this reason, pediatric nephrologists tend to treat the disease from the onset, aiming at halting the pathogenetic processes, based on expert opinion and general confidence with steroids for the lack of large pediatric controlled studies. Glucocorticosteroids are widely used, although without full comprehension of the fine molecular effects on IgAN, mostly based on trials performed in adults. In this review, a critical analysis of adult data is provided for extrapolating information useful for children, with a parallel evaluation of the results of the TESTING Trial, employing oral methylprednisolone, and of the NEFIgArd Trial, using enteric release budesonide. Patients’ characteristics and the scheme of the two studies are surprisingly similar: Nefecon and methylprednisolone showed 40–50% proteinuria reduction from baseline, with a fast effect of methylprednisolone (3–6 months) and a similar effect on renal function decline. Large genome-wide studies, above-risk alleles, also discovered risk loci targetable by multiple drugs particularly those involved in the modulation of the mucosal immunity priming of B-cells toward the production of galactose deficient IgA1 (Gd-IgA1). The new KDIGO 2024 guidelines under public review in recent months will lower the proteinuria threshold for treatment to 0.5 mg/mg and consider the value of Nefecon in reducing the levels of Gd-IgAI1. The choice between old and new corticosteroids in treating children with IgAN is approaching. In the near future, the genetic data, complemented by blood and urine biomarkers, could be included in tools to guide therapeutic choices and monitoring.

**Graphical Abstract:**

**Figure:**
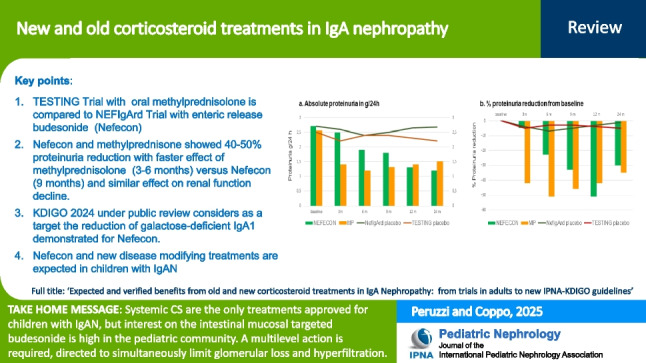
A higher resolution version of the Graphical abstract is available as Supplementary information

**Supplementary Information:**

The online version contains supplementary material available at 10.1007/s00467-025-06725-1.

## Introduction

IgA nephropathy (IgAN), a unique pathology entity defined by glomerular deposits of immunoglobulin A (IgA), dominant or co-dominant over the other classes of immunoglobulins, rarely progresses to Stage 5 chronic kidney disease within childhood [1]. However, IgAN is a chronic disease, and, albeit spontaneous remissions are possible in mild cases [2], children are at risk of progression over decades of adult life. Children with IgAN present with a trajectory of eGFR with an initial phase after biopsy of kidney function improvement, due to benefits of treatment or disease spontaneous self-limitation of the acute inflammation [3]. Subsequently, eGFR in children has a plateau phase followed by linear decline, like in adult patients. Hence, IgAN in children is recognized as a disease requiring expert care, since acute phases may show a temporary recovery but can have a subtle pauci-symptomatic progression to irreversible kidney histological changes and permanent loss of kidney function. On the other hand, unnecessary exposure to potentially toxic drugs should be avoided, particularly in children.

Glucocorticosteroids (CS) have been the milestone of the treatment of IgAN over decades, although the fine molecular mechanisms targeting the pathogenetic factors involved in IgAN are only partially known. Indications in the past century and KDIGO 2012 suggested a 6-month course of CS in adults and children with persistent severe proteinuria (> 1 g/day) despite 3–6 months of renin–angiotensin system blockade (RASB) [4]. Protocols in children were derived from adult studies, as three intravenous pulses of methylprednisolone 1 g on months 1, 3, and 5 and oral prednisone (0.5 mg/kg on alternate days), for a total of 6 months [5] or oral prednisone 0.8–1 mg/kg/day for 2 months, tapered over 6 months [6]. These randomized controlled trials (RCTs) were performed more than 20 years ago, without complete RASB, since this treatment was not routinely used, and with mild surveillance of adverse events in both treated and placebo arms, based on the confidence of decades of experience on CS in various diseases. However, both the therapeutic efficacy and safety profile of systemic CS adopting the previous protocols for IgAN were questioned by new millennium RCTs [7–9] which associated strict treatment with RASB and took into great consideration the adverse events.

## STOP-IgAN and TESTING trials

The STOP-IgAN trial used two corticosteroid–immunosuppressive (CS/IS) regimens according to eGFR for 3 years (patients with eGFR > 60 ml/min received the methylprednisolone pulse protocol, while patients with eGFR < 60 mL/min received oral prednisone and cyclophosphamide followed by azathioprine), compared with full supportive care (SUP), based on rigorous RASB and various lifestyle health measures [7]. In the methylprednisolone pulse arm of the protocol, more patients than in the SUP arm reached the end point of complete proteinuria remission (< 0.3 g/day), but without effects of eGFR protection. During the RCT, a high frequency of adverse events was reported by meticulous safety surveillance in both CS/IS (40% of patients) and placebo (36% of cases) arms. A few severe adverse events (SAE)—impaired glucose tolerance and obesity—were significantly more frequent in the CS/IS group. The frequency of SAEs of infections was similar in the CS/IS and SUP groups, but the authors were worried about one death due to *Pneumocystis* sepsis in the treatment group, suggesting caution for infections. Moreover, 10 years after the end of the STOP-IgAN RCT, no long-term benefits were observed in the 40% decline in eGFR or stage 5 chronic kidney disease (CKD 5) [10]. No difference in adverse events was reported in the two groups. The conclusion was that, despite strenuous SUP care, 50% of STOP-IgAN trial patients after 8 years reached the endpoint (40% eGFR loss or CKD 5) independently from the association of CS/IS with RASB.

The Therapeutic Evaluation of Steroids in IgA Nephropathy Global (TESTING) RCT used the oral CS protocol (0.6–0.8 mg/kg/day of methylprednisolone tapered over after 6–9 months) versus RASB (placebo) [8]. Notably, the RCT was discontinued during recruitment because of excess SAEs (mostly infections, including two deaths) in patients receiving CS (14.7% in the methylprednisolone group versus 3.2% in the placebo group, *P* = 0.001). Albeit the preliminary results suggested the benefits of CS in proteinuria and eGFR decline, the early termination of the RCT did not allow any conclusion.

These studies focused great attention on the potential side effects and limited benefits of systemic CS in adults with IgAN. Based on these RCTs, KDIGO 2021 concluded the uncertainty over the safety and efficacy of CS treatment for IgAN and suggested a detailed discussion of the risks and benefits, particularly for patients with eGFR < 50 ml/min as well as the possibility of entering a trial with new experimental drugs [11]. After these considerations, KDIGO 2021 suggested a 3–6 month course of CS only in patients remaining at risk for progression with persistent proteinuria > 1 g/day despite maximal supportive care including a low-sodium diet and maximally tolerated doses of RASB [11]. These indications were extended to children as well, but a KDIGO practice point reported that many pediatric nephrologists usually treat children with proteinuria > 1 g/day or UPCR > 1 g/g and/or mesangial hypercellularity, with CS in addition to RAS inhibitors immediately after renal biopsy [12].

Recent advancements on systemic CS were provided shortly after KDIGO 2021, by the final TESTING RCT [9]. In this study methylprednisolone dose was reduced from 0.6 to 0.8 mg/kg/day for 2 months (max. 48 mg/day), tapering over 6–8 months to 0.4 mg/kg/day for 2 months (max 32 mg/day), and the prophylaxis for *Pneumocystis jirovecii* was added for 3 months. The study involved 257 CS-treated patients (140 with full doses and 117 with low doses) and 246 patients on placebo (RASB). Most patients were of Chinese ethnicity (76%), and 5% only were European patients. After 4.6 years a significant effect on the primary endpoint (40% reduction GFR or CKD 5) was observed in 74 patients (28.8%) in the methylprednisolone group compared with 106 (43.1%) in the placebo group (*P* < 0.001), and low doses were found to be as effective as full doses. SAEs were reported in 10.9% of treated patients versus 2.8% in the placebo arm. In the methylprednisolone group, reduced doses of SAEs were reported in six cases versus three in the placebo group, with infections in three cases (one fatal). In the methylprednisolone group, full-dose SAEs were reported in 22 cases versus four in the placebo group, with infections in 17 cases (3 fatal). Hence, the conclusion was on the efficacy of methylprednisolone treatment in IgAN with persistent proteinuria despite careful RASB, but with the warning of side effects, particularly infections.

## NEFIGAN and NefIgARD trials

An innovative disease-modifying approach to CS therapy in IgAN was offered by the use of an oral CS formulation designed to target the gut-associated lymphoid tissue at the Peyer’s patches, a major site of production of galactose-deficient IgA1 (Gd-IgA1), thought to be the first initiating event in the pathogenesis of IgAN. This is of theoretical interest also for limiting the systemic CS adverse effects.

A formulation of budesonide (Nefecon) was designed for drug delivery at the distal jejunal site near the ileocecal junction where the Peyer’s patches are most abundant. Budesonide is a powerful CS with a formula that favors high local anti-inflammatory activity [13–15]. Moreover, it has a high hepatic clearance, as 90% of the drug is cleared at its first liver passage. Budesonide has been used for local anti-inflammatory effects in inflammatory bowel diseases and asthma [14], in adults and children [15, 16]. Nefecon has a peculiar coating that favors the active drug delivery near the ileocecal junction, the site of the greatest presence of Peyer’s patches and polymeric IgA rich in Gd-IgA1 production.

The first indications of potential benefits of Nefecon in IgAN patients came from the NEFIGAN a phase IIb RCT [17]. Patients with persistent proteinuria despite optimized supportive care treated for 9 months with Nefecon had a significant reduction in proteinuria (− 27.3% in patients on 16 mg/day versus + 2.7% in placebo) with stable eGFR.

The NefIgArd Phase II/III study was addressed to verify the efficacy and safety of Nefecon 16 mg/day versus placebo in patients with IgAN and proteinuria > 0.8 g/g or > 1 g/24 h despite supportive care. Part A results involved 199 patients receiving Nefecon or a placebo for 9 months and followed for an additional 3 months [18]. After 9 months of RCT, Nefecon-treated patients had a 27% reduction in UPCR compared to those in placebo (*P* < 0.0003), and eGFR was significantly preserved (difference versus placebo 3.87 ml/min/1.73 m^2^, *P* = 0.0014). Treatment-emergent adverse events (TEAEs) were mild or moderate, severe in intensity in 1% of the cases. These efficacy results led to conditional accelerated approval by the U.S. Food and Drug Administration (FDA) of TARPEYO (budesonide) delayed-release capsules for reducing proteinuria in patients with IgAN.

Complete follow-up to assess the efficacy of kidney function was reported in Part B NefIgArd study: a total of 364 patients were randomized to Nefecon 16 mg or SUP [19]. Patients were stratified according to baseline proteinuria (< 2 or ≥ 2 g/24 h), baseline eGFR (< 60 or ≥ 60 ml/min/1.73 m^2^), and region (Asia–Pacific, Europe, North America, and South America). Most patients (76%) were White. Patients were followed for 9 months of treatment with Nefecon and 15 months of observational follow-up period of the study drug. During the whole period, patients and investigators were blinded to treatment assigned by randomization. Optimized SUP was continued over the 2 years. The primary efficacy endpoint considered a time-weighted average of eGFR over 2 years. The eGFR benefit observed at 9 months was maintained for 15 months without treatment. Time-weighted average eGFR loss over 2 years was − 2.5 ml/min/1.73 m^2^/year in the Nefecon group versus − 7.5 ml/min/1.73 m^2^/year in the placebo group (*P* < 0.0001) with a gain in the total slope of 2.9 ml/min/1.73 m^2^/year (*p* < 0.0001) in favor of Nefecon. This was considered as 50% less deterioration in kidney function in Nefecon-treated patients than in SUP care over the 2-year period.

The effect on eGFR decline was similar in patients with baseline UPCR below 1.5 g/g or higher, and this satisfied a concern of the results in Part A of the study where the benefits seemed to be of non-significant relevance in patients with UPCR below 1.5 g/g. Percentage changes in UPCR showed a median 30% reduction after 9 months of treatment, with a further reduction at 12 months (47%). From 9 to 24 months of treatment, proteinuria reduction was maintained at around 40% compared to placebo. Notably, at the end of 2 years, the dipstick was negative for microscopic hematuria in 59% of the Nefecon group versus 39% of the placebo group.

During 9 months of treatment, Nefecon has a good safety profile. Treatment-emergent SAEs were reported in 10% in the Nefecon group versus 5% in SUP. Compared to the placebo, the Nefecon group had more hypertension (17%), peripheral edema (12%), muscle spasms (12%), and acne (11%). These AEs were considered non-serious and of mild severity. Serious AE-related infections were reported in 3% of patients on Nefecon and 1% in the placebo group. Treatment-emergent AEs leading to the discontinuation of the study treatment were reported in 9% of patients in Nefecon and 2% in placebo groups. During the follow-up period, the incidence of AE was similar in the two groups. In December 2023, the FDA approved budesonide delayed-release capsules to reduce the loss of kidney function in adults with primary IgAN at risk for disease progression**,** followed by EMA authorization in 2024.

## Parallel evaluation of effects on proteinuria and kidney function of systemic CS (TESTING RCT) and budesonide targeted release formulation (NEFIgArd RCT)

We aimed to provide an easy approach to evaluate the results obtained in adult patients with IgAN treated with 9 months of Nefecon and 6–9 months of oral methylprednisolone described in NefIgArd and TESTING RCTs. A comparison of these results should be interpreted with the limitation that the two trials enrolled different populations and had different designs; however, careful analysis of the reported data offers a comprehensive evaluation. Details on patients’ baseline data, proteinuria absolute values, and percentage changes during treatment and follow-up were captured from Tables and Figures published in the text and in online supplements of each publication.

**Fig. 1:**
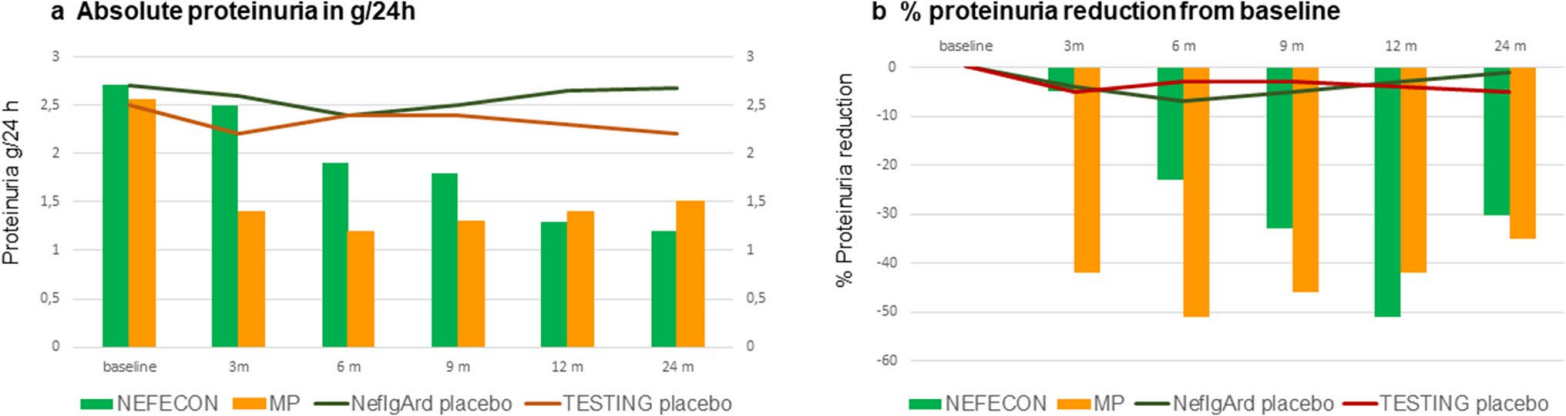
Comparison of efficacy between targeted-release formulation budesonide Nefecon in the NefIgArd trial and systemic corticosteroids in the TESTING trial

Despite the difference in patients’ demographic data, mostly of the prevalence of ethnicity (76% White in NefIgArd versus 76% Chinese in TESTING), the median values at baseline of proteinuria and GFR were surprisingly very similar (Table 1 and Fig. 1).

**Table 1:** Parallel evaluation of efficacy between targeted-release formulation budesonide Nefecon in the NefIgArd trial and systemic corticosteroids in the TESTING trial

	**NefIgArd** [19]		**TESTING** [8]	
**Investigational drug**	Nefecon 16 mg/day, placebo		Methylprednisolone 0.8–0.4 mg/kg/day, placebo	
**Number of patients**	182 Nefecon; 182 placebo		136 MP; 126 placebo	
**Baseline proteinuria**, g/24 h	Mean, 2.71 ± 1.73; median, 2.29 (IQ, 1.61–3.14)		Mean 2.55 ± 2.45; median, 1.99 (IQ, 1.36–3.09)	
**Proteinuria reduction**	**% Reduction from baseline**	**Absolute proteinuria g/24 h**	**% Reduction from baseline**	**Absolute proteinuria g/24 h**
6 months	− 17%	1.9	− 50%	1.2
9 months	− 30%	1.8	− 48%	1.3
12 months	− 50%	1.35		
24 months	− 40%	1.2	− 37%	1.5
**Baseline GFR**, ml/min/1.73 m^2^	median 56.1 (45–70)		56.1 (43–75)	
**GFR modifications**	**Absolute GFR variation from baseline, ml/min/1.73 m**^**2**^	**Absolute GFR, ml/min/1.73 m**^**2**^	**% GFR variation from baseline**	**Absolute GFR, ml/min/1.73 m**^**2**^
3 months	+ 2.7	59	+ 10%	65
6 months	+ 1.2	57.2	+ 10%	65
9 months	+ 0.6	56.6	+ 5%	64
12 months	− 1.5	54.5	+ 3%	63
24 months	− 6.1	50	+ 1%	60

### Effects on proteinuria

Baseline proteinuria values were mean 2.71 ± 1.73 g/24 h (median values 2.29 (IQ 1.61–3.14 g/24 h)) in 182 patients treated with Nefecon 16 mg and 2.55 ± 2.45 g/24 h (1.99 (IQ 1.36–3.09 g/24 h)) in 136 patients treated with methylprednisolone.

In Nefecon-treated patients, proteinuria showed a reduction of 17%, 30%, and 50% at 6, 9, and 12 months, respectively, maintaining a 40% reduction (values at around 1.2 g/24 h) at 24 months (Table 1 and Fig. 1).

In patients treated with methylprednisolone (0.4 to 0.8 mg/kg/day), proteinuria showed a 50% reduction in 3–6 months from 2.5 to 1.2 g/24 h; proteinuria was almost stable at 9–12 months (1.3 g/24 h) with 48% reduction from baseline. During the following year, proteinuria had a mild increase to values of 1.5 g/24 h (a decrease from a baseline of 37%) (Table 1 and Fig. 1).

In conclusion, methylprednisolone showed a fast antiproteinuric effect; however, the maximal antiproteinuric effect was superimposable in the two treatment protocols (40–50% proteinuria reduction). The long-lasting effects of both treatments at 1–2 years were similar, with a 40% reduction from baseline proteinuria values, maintaining persisting proteinuria of 1.2–1.5 g/24 h in both RCTs.

### Effects on kidney function

Baseline GFR values were very similar, with a median GFR of 56.1 (45–70) ml/min/1.73 m^2^ in 182 patients treated with Nefecon 16 mg, and 56.1 (43–75) ml/min/1.73 m^2^ in 136 patients treated with methylprednisolone.

In patients treated with Nefecon at 3 months, a GFR increase of + 2.7 ml/min was detected (GFR, 59 ml/min/1.73 m^2^; positive difference from placebo, 4.8 ml/min/1.73 m^2^), of lesser extent at 6 months with a GFR increase of + 1.2 ml/min/1.73 m^2^ (GFR 57.2 ml/min/1.73 m^2^, difference from placebo 4.5 ml/min/1.73 m^2^), and with almost no difference from baseline at 9 months when GFR was similar to initial values (Table 1 and Fig. 1). At 12 months, a slow decline in GFR was observed with GFR − 1.5 ml/min/1.73 m^2^ in comparison to baseline. The decline was more evident, similar to placebo at 24 months (Table 1 and Fig. 1).

In patients treated with methylprednisolone at 3 months, the median GFR was 65 ml/min/1.73 m^2^, with an absolute change of + 10%, which remained stable at 6 months and slightly lower at 9 months, when GFR was 64 ml/min/1.73 m^2^, and almost unchanged from baseline at 12 months. GFR values remained stable at 24 months (GFR 60 ml/min, similar to baseline and difference from the placebo in GFR of 7 ml/min/1.73 m^2^) (Table 1 and Fig. 1).

In conclusion, in the two independent RCTs Nefecon and methylprednisone showed similar increases in GFR at 3–6 months and a parallel GFR decline over 2 years. Patients in the Nefecon group had a loss of GFR of − 6 ml/min/1.73 m^2^ while the placebo had − 12 ml/min/1.73 m^2^ over 2 years of follow-up. Patients on methylprednisolone had a GFR loss of − 2.5 ml/min/1.73 m^2^/year while the placebo had a loss of − 4.9 ml/min/1.73 m^2^/year over 3.5 years in median follow-up.

## Inference from genetic studies on therapeutic choices

IgAN is a non-Mendelian disease with strong genetic conditioning demonstrated through large genome-wide association studies (GWAS), predominantly within European and East Asian population cohorts.

The first output from GWAS methodology was the identification of risk alleles for the disease: over 30 loci were identified encompassing regions of the major histocompatibility complex (MHC), genes implicated in mucosal IgA production, components of innate and adaptive immunity, inflammatory responses, cytokine ligand–receptor interactions alongside the complement system [20–22]. These findings fit with multiple steps of the “multi-hit” pathogenic hypothesis [23] and confirm the role of under-galactosylated IgA1 (Gd-IgA1) as triggering hit of IgAN, previously demonstrated to be a heritable trait [24, 25] although not sufficient to induce the disease by itself.

Subsequent focused GWAS studies demonstrated that the defective galactosylation of IgA is genetically determined by several loci [26] among which the two loci *C1GALT1*, encoding Core-1-synthase-glycoprotein-*N*-acetylgalactosamine-3-β-galactosyltransferase, a galactose transferase crucial for generating normal *O*-glycans at the hinge region of circulating IgA1, and *C1GALT1C1*, coding for Cosmc, a molecular chaperone required for proper folding in the endoplasmic reticulum and to provide stability and full functionality of C1GALT1C, explain approximately 7% of the variability of the amount of circulating Gd-IgA1 in Europeans, but only 2% in East Asians. Analogous conclusions were obtained in an independent cohort from the UK of both White and Asian origins [27].

A recent large GWAS study aimed at exploring new IgAN risk alleles enrolled 38,897 individuals (10,146 biopsy-proven IgAN cases and 28,751 controls) across 17 international cohorts of European and East Asian ancestry. This study identified 30 independently significant risk loci, some of them within the HLA region and 24 non-HLA [22]. Sixteen risk loci were not previously reported and coded for genes relevant to immune regulation, cellular proliferation, and mucosal immune system.

The novelty of this recent analysis is the shift from the consideration of risk alleles to the exploration of candidate genes with the aim of identifying new drug-targetable mechanisms.

**Fig. 2:**
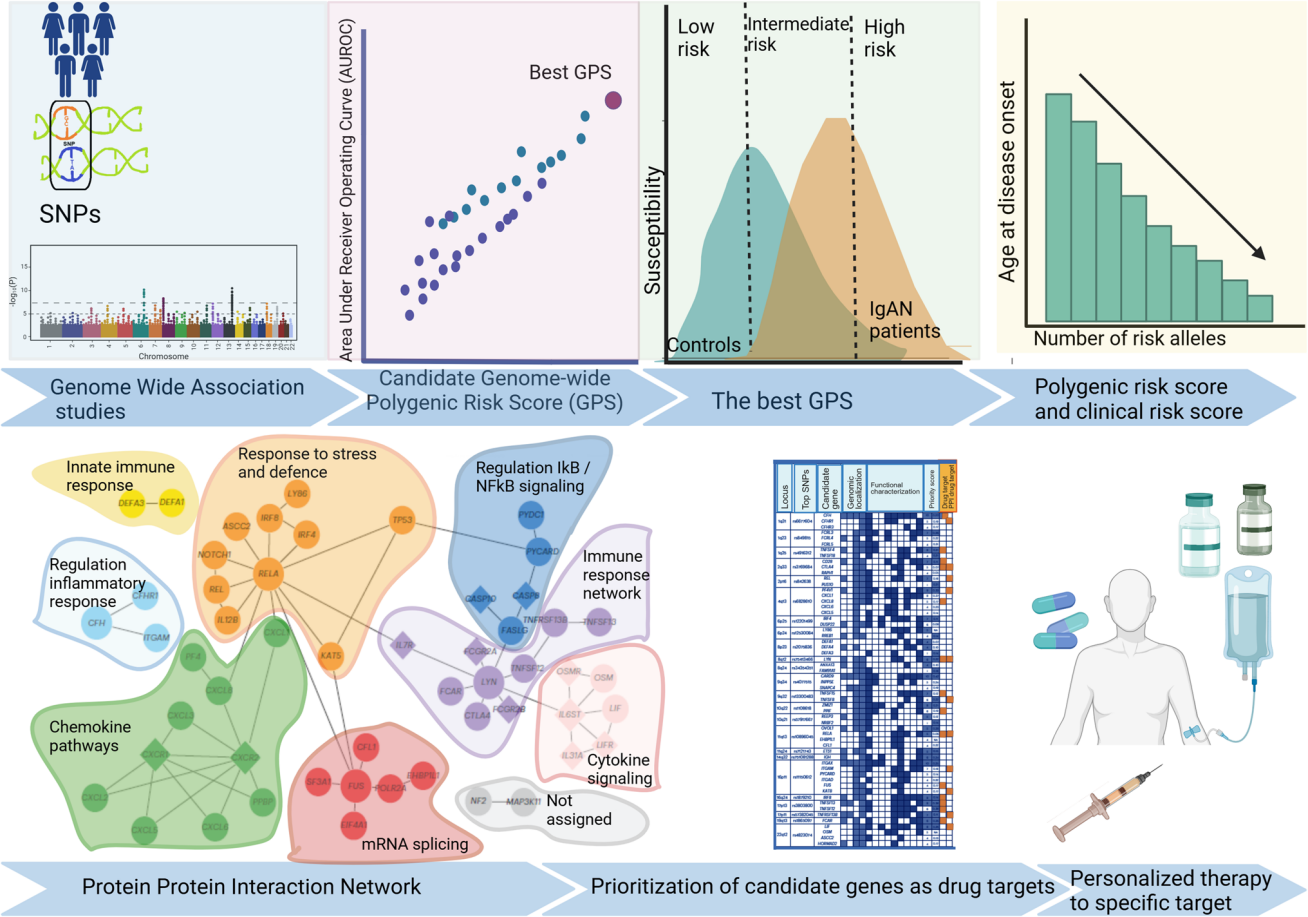
The evolution of genetic studies in IgAN: from genetic risk loci to polygenic risk score and functional annotation of gene candidates for encoding therapeutic targets for new drugs. The figure represents the evolution of genetic studies in IgAN, from the identification by genome-wide studies of single-nucleotide polymorphisms representing risk alleles, to the studies in large cohorts of different ethnicities to provide the best candidate genome-wide polygenic risk score, able to discriminate low, intermediate or high risk of disease susceptibility, to the combination of genetic risk score and clinical risk scores influencing the age of disease onset. In recent studies, Protein–Protein interaction network analysis was able to identify multiple systems involved in the pathogenesis of IgAN. Among these proteins, functional studies were able to identify candidate targets of new specific drugs [22]. Future expectations convey the idea of a genetic risk score combined with clinical risk scores provided by prediction tools to identify the best personalized therapeutic approach. SNPs single-nucleotide polymorphisms, GPS genome-wide polygenic risk score; the figure was created in https://BioRender.com

Through the analysis of protein–protein interactions, a network comprising 76 candidate proteins exhibited enrichment in multiple mechanisms: stress and defense pathways, immune response, cytokine-mediated signaling, and regulation of nuclear factor kappa B (NF-κB) signal transduction (Fig. 2).

Several key points of these pathways can be targeted by glucocorticoids, acting through the engagement of the glucocorticoid receptor (GR), which translocates to the nucleus and regulates the transcription of multiple target genes, by binding to consensus DNA sequences, namely, glucocorticoid response elements, present in the enhancer and promoter regions of GR target genes (reviewed in the study of Strickland et al. [28]). Glucocorticoids are therefore able to regulate a large number of responsive genes either inducing activation or repression of transcription, and these pleiotropic effects explain the many positive but also the undesired effects.

From Kiryluk et al.’s [26] study, the initial pool of 308 candidate genes was functionally prioritized, meaning it was assessed whether any of these regions encoded a protein that directly interacted with a pharmacologically active drug target approved or in development for human diseases. Out of the total, 13 GWAS regions encompassed 17 proteins already targeted by existing drugs, while 11 regions included 14 proteins with direct protein–protein interaction targets.

One of the most relevant results was the identification of ligand–receptor pairs, such as APRIL and its receptor TACI, encoded by two distinct significant loci, *TNFSF12/13* and *TNFRSF13B*, respectively, strictly involved in the mucosal triggering mechanisms and targeted by specific drugs subsequently demonstrated to be highly effective in IgAN [29, 30].

Moreover, from this study, the identification among the risk alleles of Complement Factor H (CFH), a strong regulator of the alternative complement pathway, further strengthened the rational use of complement inhibitors targeting the alternative complement pathway recently investigated in clinical trials in IgAN with interesting results [31–33].

Other interesting loci offer targets for drugs inhibiting T-cell activation by targeting CD28 ligands, inhibitors of IL-8 or its receptor, and inhibitors of the nuclear factor-κB pathway.

Some of the top-priority genes currently lack available drug treatments (*CARD9*, *ITGAX*, *PF4V1*, *CFHR1*, *FCAR*), thus presenting promising prospects for the development of novel therapeutics aimed at addressing IgAN. Furthermore, certain loci encode secreted proteins, such as FCRL3 and TNFSF4, which may have a protective role, suggesting that targeting their upregulation could be a valuable therapeutic strategy.

Based on GWAS output a genome-wide polygenic risk score (GPS) was set up and assayed on independent cohorts where the algorithm was able to explain up to 11% risk of disease. Interestingly, the new GPS confirmed the data obtained in the first study on 15 SNPs [21] with GPS inversely associated with the age at diagnosis. The GPS was also significantly associated with hematuria. Other studies explored the genetic risk score associated with IgAN progression in different populations and using different combinations of SNPs, suggesting that incorporating genetic risk factors could enhance the predictive performance of clinical-pathological risk models but possibly in the near future could be included in tools to drive therapeutic choices [34].

In conclusion, IgAN is modulated by genes regulating both the innate and the adaptive immune response as well as the inflammatory cascade, albeit none can be considered as specifically targeted only by systemic or mucosal-targeted corticoids.

## Effects on biological markers of Nefecon, a CS formulation designed to target the gut-associated lymphoid tissue

Along with the definition of the multiple steps of immunological pathogenetic mechanisms and the exploration of several drugs targeting these key points, a recent impulse was given to the search for biomarkers able to catch the modifications induced by various treatments.

Some traditionally known parameters checked in patients with IgAN, such as total serum IgA, serum Gd-IgA1, and complement fraction C3 alone or as IgA/C3 ratio and Gd-IgA/C3 ratio, are being re-evaluated in long observational studies as predictors of progression, but still scarcely used within therapeutic clinical trials (reviewed in [35]).

**Fig. 3:**
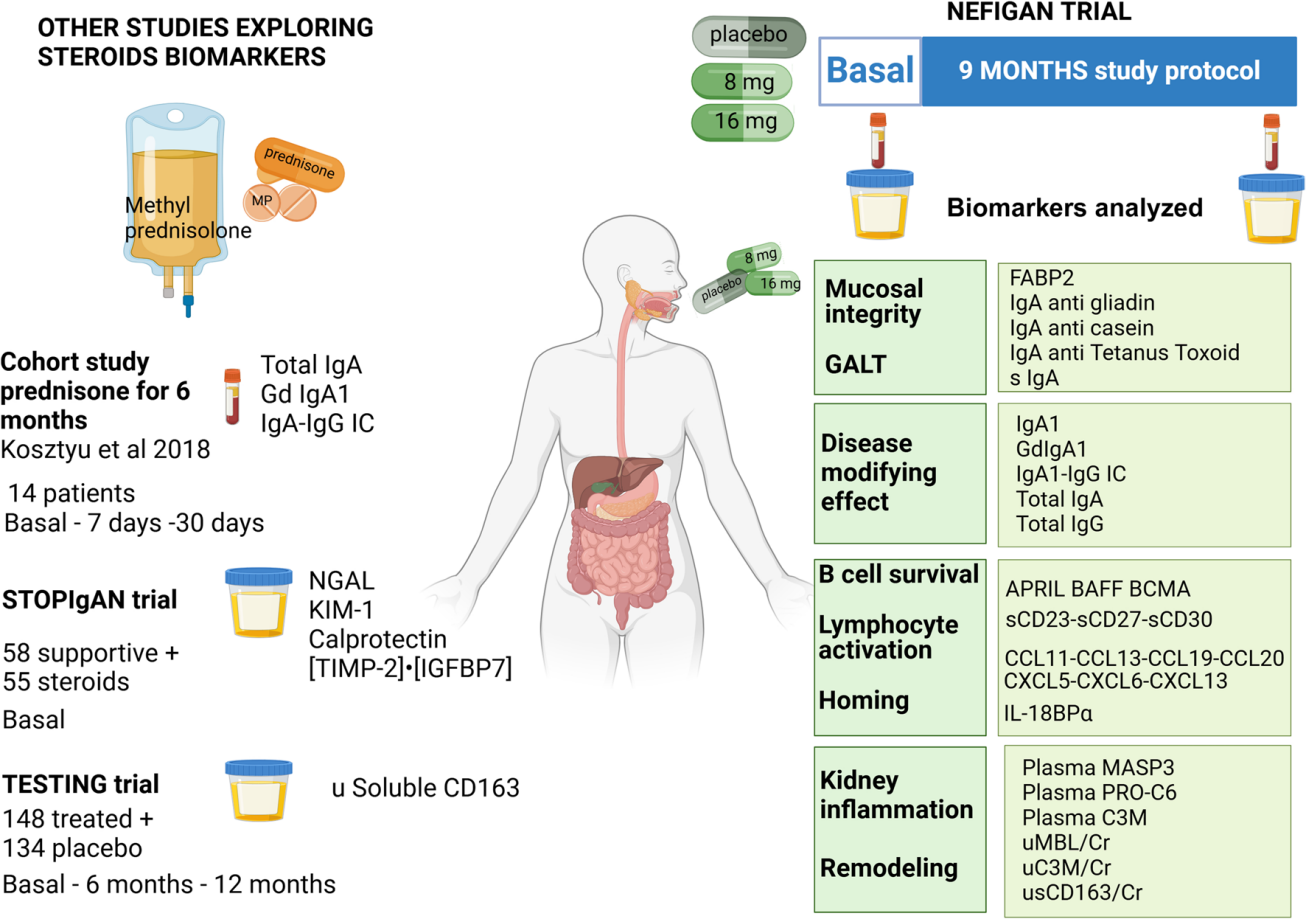
Biomarkers explored in corticosteroid trials. The figure represents the results on biomarker studies in clinical trials exploring systemic glucocorticosteroids: prednisone cohort study Kosztyu et al. [37], STOP-IgAN TRIAL biomarker study [38], TESTING TRIAL biomarker study [39], and the results obtained exploring several biomarkers in the NEFIGAN study [36] on Nefecon treatment. FABP, fatty acid-binding protein 2; GdIgA1, galactose deficient IgA1; APRIL, A proliferation-inducing ligand; BAFF, B-cell activating factor; BCMA, B-cell maturation antigen; CCL, C–C motif chemokine ligand; CD, cluster of differentiation; CXCL, C-X-C motif chemokine ligand; IL, interleukin; IL-18BPa, interleukin 18 binding protein isoform alpha; MASP3, plasma mannose-binding lectin-associated serine protease 3; C3M, a fragment of type-III collagen released by matrix metalloproteinase-mediated extracellular matrix degradation; PRO-C6, propeptide of type-VI collagen; uMBL, urinary mannose-binding lectin; uC3, urinary complement C3 fraction; uNGAL, urinary neutrophil gelatinase-associated lipocalin; KIM-1, kidney injury molecule-1; [TIMP2•IGFBP7], the product of tissue inhibitor of metalloproteinase-2 and insulin-like growth factor-binding protein 7; u Soluble CD163, urinary-soluble cluster of differentiation 163. Figure was created in https://BioRender.com

A large set of immunological and inflammation biomarkers was included in the NEFIGAN trial (NCT01738035) [17], and the results of the modifications induced by a 9-month course of treatment with Nefecon were recently published [36]. In total, 150 patients were enrolled, stratified on their baseline urine protein–creatinine ratio (≤ 0.9 g/g and > 0.9 g/g) and randomly assigned in a 1:1:1 ratio to receive either Nefecon 8 mg/day, Nefecon 16 mg/day, or placebo, respectively. Following a 6-month run-in phase, patients underwent a 9-month treatment period. Plasma and urine samples were collected at baseline, at the end of the treatment phase, at 9 months, and at 12 months during the follow-up phase (Fig. 3).

In this study, the biomarkers analyzed were selected, according to the huge amount of biochemical and genetic data available, for exploring with different methodologies the effects induced by enteric-coated budesonide on local immunological dysregulation of IgAN, phlogistic response, regeneration of tissues, and complement control.

Traditional biomarkers of IgAN (total IgA, secretory IgA, IgA-IgG immunocomplexes, Gd-IgA) were added to an additional panel aimed at exploring intestinal mucosal integrity, as intestinal-type fatty acid-binding proteins (FABP2 and FABP6) and a large panel of markers of B-cell survival (B-cell activating factor (BAFF)/B-lymphocyte stimulator/tumor necrosis receptor superfamily member 13B (TNFRSF13B) or APRIL, B-cell maturation antigen/TNFRSF17), of lymphocyte activation and of immune response (soluble CD23, CD27, CD30, and antibodies against common antigens, such as tetanus toxoid). Moreover, mucosal homing, inflammation (a panel of C–C and C-X-C chemokines), tissue remodeling (plasma and urine sCD163, collagen type-III degradation marker (C3M), and propeptide of type-VI collagen) were included.

Protein–protein interactions were explored in the STRING database, Gene Ontology, and Kyoto Encyclopedia of Genes and Genomes (KEGG) pathways as sources for annotations.

The results are impressive, since, for the first time, the parameters chosen to investigate the potential biological effects induced by an investigational drug were able to demonstrate that enteric-coated budesonide hits the initial mucosal origin of the pathogenetic cascade. In detail, Nefecon induced both direct effects on the intestinal mucosa, reducing the release of the integrity marker FABP2, and immunological effects on IgA-producing plasma cells, as witnessed by the decrease of serum total IgA and secretory IgA but also by the reduction of anti-gliadin and anti-casein circulating IgA. Moreover, the reduction upon treatment of IgA–IgG immunocomplexes, of APRIL, BAFF, BCMA, sCD23, sCD27, sCD30, and of a large set of intestinal expressed chemokines, support the hypothesis that enteric-coated budesonide can be considered a disease-modifying drug, in its capacity to directly address hits 1, 2, and 3 of the pathogenetic cascade directly in the gut-associated lymphoid tissue.

The data on the effects induced by enteric-coated budesonide are encouraging in their conclusive demonstration of a disease-modifying effect directly on the gut-associated lymphoid tissue and the possibility to modulate therapy according to the reduction of biological parameters expressing the activation of the initiating phenomena, such as APRIL/BAFF serum concentration.

Systemic oral or intravenous corticosteroids, although widely used for decades in IgAN, were not rigorously and extensively investigated in their ability to modify specific biomarkers of disease and systematic data have not been rigorously collected so far.

In a small study on 14 IgAN patients with persisting proteinuria > 1 g/24 h and normal renal function treated for 6 months with oral prednisone 60 mg/day/m^2^, a significant reduction of total IgA and Gd-IgA1, although not reaching normalization, was observed in parallel with proteinuria decrease. In this study, IgA sialylation was not modified by prednisone [37].

In the STOP-IgAN trial, a subset of 113 participants had urine samples collected at the end of the run-in phase available for assay of urinary NGAL, KIM-1, calprotectin, and [TIMP-2]•[IGFBP7] as an expression of tubulointerstitial damage and intestinal inflammation. All biomarkers did not prove predictive of reaching the end point of full clinical remission, eGFR loss ≥ 15 and 30 ml/min/1.73 m^2^ over the 3-year trial phase of STOP-IgAN, either in subjects receiving only supportive therapy or immunosuppression [38]. These biomarkers therefore are not of help in deciding whether to start systemic steroids or immunosuppression, and neither can they be used as parameters of response since they were not monitored during the treatment phase.

In the TESTING trial, a subset of 282 patients (134 placebo and 148 treated) had urine samples available at baseline, at 6 months, and at 12 months of treatment: in these subjects, soluble urinary CD163, a marker of macrophage infiltration, displayed a good correlation with active histology lesions, lower eGFR, and higher degree of proteinuria at baseline and displayed a significant reduction after initiating methylprednisolone treatment at 6 and 12 months compared to placebo (*P* < 0.001), independently from the methylprednisolone dose administered. This marker was evaluated only in the Chinese cohort and was followed by a reduced time schedule but offers significant stimuli for further development and exploration under more stringent conditions in other RCTs [39].

For the time being, none of the novel biomarkers investigated in IgAN, including those derived from omic sciences, such as proteomics and metabolomics, and advanced techniques of single-cell RNA expression studies, has been validated in large cohorts as an expression of drug-induced modifications. The preliminary studies on the NEFIGAN and the TESTING trials are encouraging, and it is expected in the near future that genetic risk score and a panel of biomarkers can be included in the prediction tool, and even more that a panel of biomarkers can be used to monitor the medication response and drive therapeutic choices.

## IPNA clinical practice recommendations for corticosteroid treatment in children with IgAN

The recent IPNA clinical practice recommendations for the diagnosis and management of children with IgAN and IgAVN [40] consider the only formulation of corticosteroids presently allowed, i.e., systemic glucocorticosteroids. In children, on the one hand, large controlled multicentric trials on steroids have not been conducted, but on the other, pediatric nephrologists have a generally positive experience and wide consensus on the early use of steroids. Although unable to draw evidence-based conclusions on glucocorticoid indication, dose, and duration, the final suggestion was to start steroids, following the oral or the intravenous methylprednisolone schemes derived from adults, in children at risk of progression for persistent proteinuria (UPCR 0.5–1 mg/mg (50–100 mg/mmol) despite 3 months of RASB, reduced to 4 weeks of RASB if UPCR > 1 mg/mg (100 mg/mmol), and active MEST-C score (1 or more among M1, E1, and S1 with podocyte lesions, C1). A 4- to 6-month course of glucocorticoid treatment is considered, either as oral prednisolone or intravenous pulses of methylprednisolone. Conventional pediatric protocols are adopted for glucocorticoids (prednisone 2 mg/kg/day, max 60 mg/m^2^/day) for a maximum of 4 weeks followed by alternate-day dosing tapered over 5–6 months. Lower doses as indicated from the adult TESTING trial (0.4 mg/kg/day of prednisone/prednisolone (or equivalent) for 2 months, tapering over 6 months) are considered; however, no data are available in children.

IPNA guidelines suggest a prompt use of corticosteroids in children with acute onset of IgAN and declining kidney function (eGFR < 90 mL/min/1.73 m^2^) and/or PCR > 1 mg/mg with active severe MEST-C scores (2 or more of the following scores: M1, E1, and S1 with podocyte lesions, C1) and children with crescentic forms of IgAN (C2). In cases with C2, irrespective of proteinuria, the use of I.V. glucocorticoids is suggested.

## KDIGO 2024 future recommendations general outline

The KDIGO 2024 Clinical Practice Guideline for the Management of IgA Nephropathy (IgAN) and IgA Vasculitis (IgAV) was recently available for public review. The guideline is now being prepared for publication, incorporating feedback received during the public review period (KDIGO-IgAN-IgAV-Guideline-Public-Review-Draft-Data-Supplement_August-2024). KDIGO 2024 mostly focuses on the needs of adult patients at risk of progressive loss of kidney function, those with proteinuria ≥ 0.5 g/day, while on or off treatment, hence, lowering the previous proteinuria threshold, traditionally set at values above 1 g/day [4]. Moreover, the target of proteinuria reduction was reduced to values lower than 0.3 g/day, possibly needing multiple drug interventions.

The most relevant change in the patient’s approach is that IgAN patients at risk of progression should receive simultaneous treatments with a double-targeting approach: on one side aiming at preventing or reducing the immune complex-mediated glomerular damage and in parallel and on the other side aiming at limiting the consequences of the existing damage on glomerular loss and hyperfiltration.

The latter is obtained with lifestyle changes and the pharmacological treatment with RASB or dual endothelin angiotensin receptor antagonism (DEARA) and sodium-glucose cotransporter-2 inhibition (SGLT2i), FDA- and EMA-approved for adults but still not for children. To address the immune-mediated active damage, anti-inflammatory drugs are recommended, where satisfying evidence was reached for GS targeting the gut mucosal immune system (Nefecon) or systemic immunity (systemic GS).

KDIGO is in favor of intestinal mucosa-acting CS due to the convincing results of Nefecon-induced reduction of the levels of pathogenetic forms of galactose-deficient IgA1, widely accepted as the initiator pathogenetic event of IgAN.

## Final consideration of present and future GS treatment in children with IgAN

Systemic CS is the only treatment available for children with IgAN [40], but interest in the intestinal mucosal-targeted budesonide is high in the pediatric community, where budesonide has been a current treatment for asthma or several intestinal inflammatory diseases for decades [16, 41]. The Nefecon formulation is a further modification of the available intestinal release commercial budesonide preparations, with the novelty of a pill coating assuring a delivery of the active drug to a specific intestinal area—the ileocecal junction—particularly rich in Peyer’s patches. Since, in these lymphoid follicles, the B cells receive the commitments to produce IgA and GD-IgA1, and the Nefecon therapeutic target is very precise. The extension of the indications of Nefecon to children with IgAN is expected, since it promises a lower systemic exposure to steroid effects with fewer adverse events providing similar benefits on proteinuria and kidney function protection.

This review aimed at preparing pediatric nephrologists to make a well-informed choice between the two drugs, comparing benefits and side effects and long-lasting effects over the first years after treatment. The therapeutic effects of both systemic and intestinal mucosa immunity–targeted treatments vanished after 2 years in the two large trials in adults. The expected benefits in children and adults with IgAN should not be different, since the pathogenetic process is the same. The early diagnosis in children limits the observation of established sclerotic chronic damage, at variance with adults. This supports the need for early treatment avoiding irreversible changes as adopted in the pediatric nephrology clinical practice. The attitude of a prompt immunosuppressive or immunomodulating approach may expose the risk of overtreatment cases that might have spontaneous clinical remission [2]. For this reason, the availability of less harmful corticosteroid formulations/doses is much anticipated.

Hence, as suggested by the recent KDIGO update under public review, a multilevel action is required, directed to simultaneously limit glomerular loss and hyperfiltration, expression of the consequences of the existing damage, with a combination of drugs, including DEARA and SGLT2i, which will be ready for pediatric use in the near future.

The potential to include anti-TNF-superfamily-cytokines (anti-APRIL and anti-BAFF) or complement activation inhibitors in the treatment of glomerular inflammation offers an exciting prospect for managing IgAN in children. These therapies could help reduce the production of Gd-IgA1 or inflammation caused by complement activation, potentially slowing the silent progression of the disease.

## Supplementary Information

Below is the link to the electronic supplementary material.

Graphical Abstract (PPTX 145 KB)
