# Supplementary material for: Expected and verified benefits from old and new corticosteroid treatments in IgA nephropathy: from trials in adults to new IPNA-KDIGO guidelines
Source: Pediatr Nephrol. 2025 Mar 5;40(7):2121–31. doi: 10.1007/s00467-025-06725-1 (PMC12116737; doi:10.1007/s00467-025-06725-1)
Supplement: Supplementary file 1 — Graphical Abstract (PPTX 145 KB) [file 467_2025_6725_MOESM1_ESM.pptx]

## Slide 1
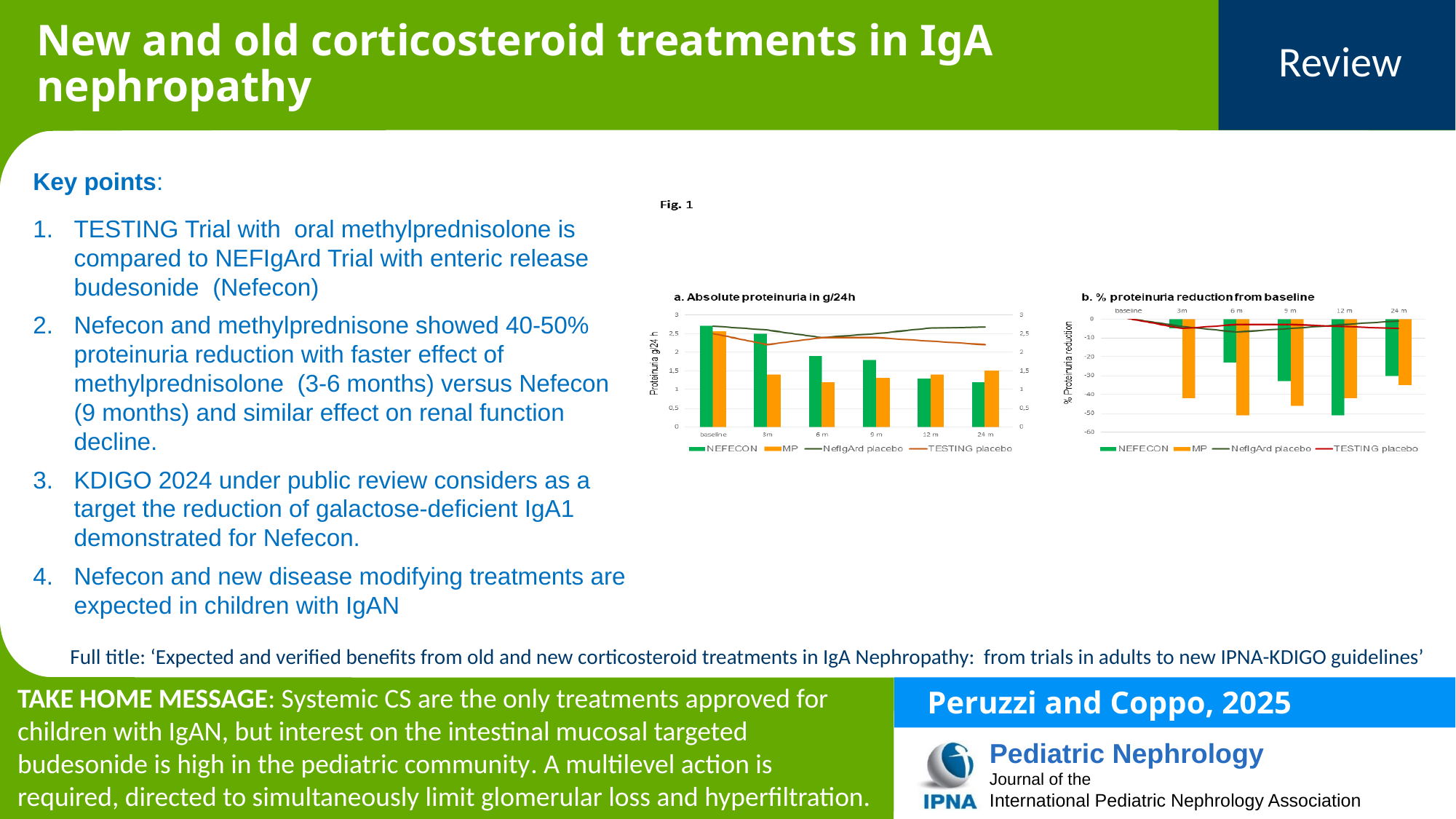

New and old corticosteroid treatments in IgA nephropathy
Key points:
TESTING Trial with oral methylprednisolone is compared to NEFIgArd Trial with enteric release budesonide  (Nefecon)
Nefecon and methylprednisone showed 40-50% proteinuria reduction with faster effect of methylprednisolone (3-6 months) versus Nefecon (9 months) and similar effect on renal function decline.
KDIGO 2024 under public review considers as a target the reduction of galactose-deficient IgA1 demonstrated for Nefecon.
Nefecon and new disease modifying treatments are expected in children with IgAN
Full title: ‘Expected and verified benefits from old and new corticosteroid treatments in IgA Nephropathy: from trials in adults to new IPNA-KDIGO guidelines’
TAKE HOME MESSAGE: Systemic CS are the only treatments approved for children with IgAN, but interest on the intestinal mucosal targeted budesonide is high in the pediatric community. A multilevel action is required, directed to simultaneously limit glomerular loss and hyperfiltration.
Peruzzi and Coppo, 2025
